# Arterial thrombosis and intracardiac thrombus as the initial presentation of a recurrent paraganglioma: case report and review of the literature

**DOI:** 10.20945/2359-3997000000342

**Published:** 2021-03-19

**Authors:** Dania Lizet Quintanilla-Flores, Jesús Zacarías Villarreal-Pérez, Claudia Analy Vélez-Viveros, Paola Portillo-Sánchez, Adriana Josefina Ortíz-Morales, José Gerardo González-González

**Affiliations:** 1 Universidad Autónoma de Nuevo Leon Hospital Universitario “Dr. Jose Eleuterio Gonzalez” Facultad de Medicina Monterrey Nuevo Leon Mexico Universidad Autónoma de Nuevo Leon, Hospital Universitario “Dr. Jose Eleuterio Gonzalez” y Facultad de Medicina, Endocrinology Service, Monterrey Nuevo Leon, Mexico

## Abstract

Pheochromocytomas and paragangliomas (PPGL) are rare neuroendocrine tumors that result in the uncontrolled release of catecholamines and secondary hypertension. They usually manifest with episodic blood pressure fluctuations, headaches and palpitations. In some cases PPGLs may be asymptomatic until they are detected as a diagnostic approach to other diseases. There have been reports that have associated PPGLs with arterial thrombosis, some with the additional finding of intracardiac thrombi. We present the case of a 21-year-old male Hispanic patient with a recurrent para-aortic paraganglioma detected by persistent hypertension, bilateral lower limb artery thrombosis and an intracardiac thrombus.

## INTRODUCTION

Pheochromocytomas and paragangliomas (PPGLs) are uncommon catecholamine secreting neuroendocrine tumors that originate from chromaffin cells, either from adrenal medulla (pheochromocytoma) or from sympathetic or parasympathetic paraganglia-associated chromaffin tissue (paraganglioma). Pheochromocytomas have an estimated annual incidence of 1–4 per million and a prevalence among hypertensive patients of 0.1–0.6%. Typically, they secrete both epinephrine and norepinephrine (1–3). Paragangliomas represent 15-20% of PPGLs and usually have a noradrenergic phenotype, with the exception of those that originate from the head and skull base that produce dopamine and its metabolite 3-methoxythyramine, or they are non-secretory (4). Recurrence rates range in 15-20% at 10 years of both PPGLs, with a 20% chances of malignancy (3).

There have been reports that have documented an association between pheochromocytoma and the development of arterial thrombosis (1,5–15), some with the additional finding of intracardiac thrombi (6,8,9,12); however, to our knowledge, a paraganglioma related to a documented arterial thrombosis has been published only in 3 patients with cerebral embolisms (16–18). We present the case of a recurrent paraganglioma detected by persistent hypertension and bilateral lower limb artery thrombosis.

## CASE REPORT

**Figure 1:**
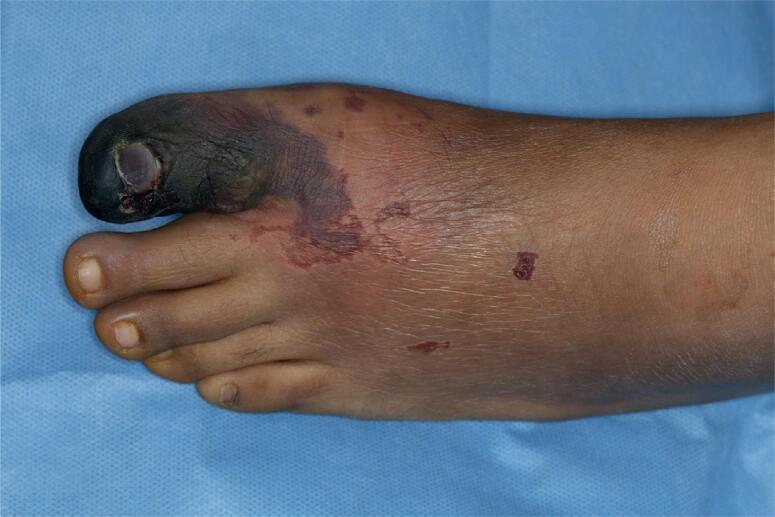
Left foot with pallor, reactive vasodilation and ischemia in the first toe

**Figure 2:**
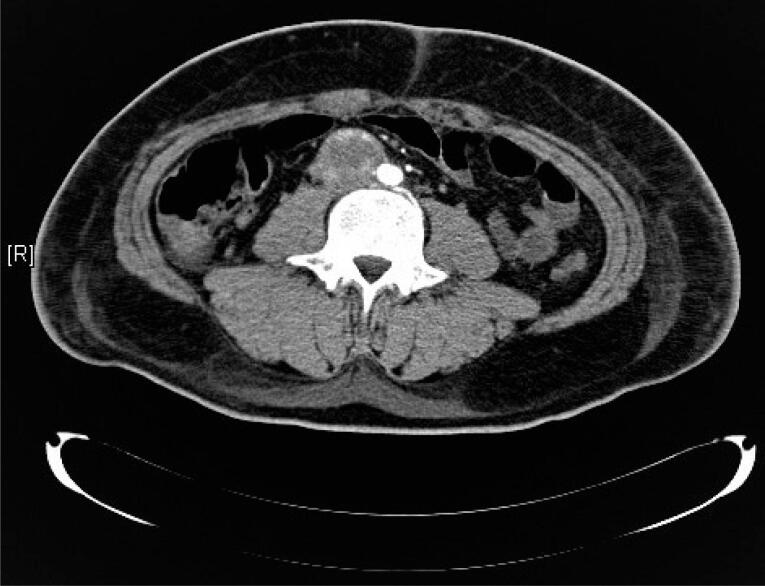
Large (5 × 5 × 5 cm) inhomogeneous para-aortic tumor, right superior to the bifurcation of the iliac arteries, between the aorta and the inferior vena cava

A 21-year-old male Hispanic patient, with a past medical history of a right para-ureteral paraganglioma diagnosed at the age of 14 and treated with surgical resection, was referred to our emergency department complaining of bilateral lower limb pain and swelling. The patient started 4 weeks earlier with lower limb edema, mostly during the night, associated with numbness, pallor, a change in coloration of the left foot and severe pain that increased with standing and walking. The foot appeared cold and pale, with a lack of tibial and pedal pulses, and ischemia of the first toe (Figure 1). When he arrived to the hospital, a hypertensive emergency was detected, requiring IV hypotensive medications. A Doppler ultrasound showed bilateral arterial thrombosis of the right femoral and left popliteal arteries. This was later confirmed by computed tomography angiography (angio-CT). Anticoagulation was started with enoxaparin. He also referred 4 months of dyspnea on exertion, orthopnea, and persistent cough. The transthoracic echocardiogram revealed a preserved ejection fraction and an intracardiac thrombus of 36.3 × 15.4 mm in the left ventricle. As an incidental finding in the angio-CT, a large (5 × 5 × 5 cm) inhomogeneous para-aortic tumor was detected, right superior to the bifurcation of the iliac arteries, between the aorta and the inferior vena cava (Figure 2). These findings led to suspicion of recurrence of the paraganglioma which imposed more specific laboratory reports. Free plasma normetanephrines were significantly elevated (1,585 pg/mL, normal 0-148 pg/mL) and free plasma metanephrines were normal (41 pg/mL, normal 0-57 pg/mL). Additional laboratory tests revealed hemoglobin of 16.7 g/dL, hematocrit 51.7%, white blood cells 8.9 × 10^3^/μL, platelets 301 × 10^3^/μL, blood glucose of 94 mg/dL and creatinine of 1.0 mg/dL. He had no family history of type 2 multiple endocrine neoplasia, neurofibromatosis, Von Hippel-Lindau disease, thyroid cancer or any other endocrine tumor. After performing a full cardio-respiratory examination and anesthetic preparation with drug blockage of adrenergic receptors, he was scheduled for an elective exploratory laparotomy and vascular embolectomy. During the procedure the 5×5×5 cm retroperitoneal tumor with infiltration to the inferior vena cava was successfully removed. The histopathology report was consistent with the diagnosis of paraganglioma. After surgery, both his blood pressure and the foot ischemia significantly improved. A control echocardiogram revealed disappearance of the intracavitary thrombus. His blood pressure remained stable during his stay in the internal medicine ward, requiring low doses of metoprolol (25 mg daily) and nifedipine (30 mg twice daily). He was discharged for outpatient follow-up, with additional warfarin as part of the therapeutic regimen of anticoagulation.

## DISCUSSION

To our knowledge, this is the first case reporting the association of a recurrent paraganglioma, peripheral lower limb arterial thrombosis and an intracavitary thrombus. Paragangliomas represent 15-20% of PPGLs. They originate from the ganglia of the sympathetic chain in the thorax, abdomen and pelvis or from the parasympathetic ganglia in the head and skull, with the abdomen being the most frequent location. They usually manifest with paroxysmal blood pressure fluctuations; however, the classic presentation includes episodic hypertension, headaches and palpitations (4). In some cases paragangliomas may be asymptomatic until they are diagnosed as a diagnostic approach to other diseases (10) as in this case, in which it happened with an arterial thrombosis in the lower limbs. Furthermore, the clinical presentation varies depending on the type of catecholamine secreted; as in our case, where the noradrenergic-mediated *α*-receptor stimulation is mainly associated with vasoconstriction, volume depletion and sustained hypertension (3).

With a reported prevalence of 16.6% (19), vascular abnormalities associated with PPGLs include renal artery stenosis, aorto-arteritis, aortic aneurysm, inferior vena cava thrombosis, stroke, and acute arterial thrombosis, with the latter being described in very few cases, and mostly associated with pheochromocytoma. Unfortunately, despite not being so infrequent, these alterations are not usually included in the main signs and symptoms associated with PPGLs, and much less are described as a reason for suspected diagnosis at the onset of the clinical presentation.

It is probable that the pathophysiology of arterial thrombosis in catecholamine secreting tumors may be multifactorial. There have been described several factors as triggers that predispose to the thrombotic events. Some of them include a prolonged and severe vasoconstriction as a result of persistent noradrenergic-mediated *α*-receptor stimulation; a chronic inflammatory state characterized by an increase in inflammation markers (C-reactive protein, fibrinogen, orosomucoid and *α*2-macroglobulin), leukocytes, neutrophil, platelet counts and cytokines (Interleukin 6) (11,20); an association with autoimmune diseases leading to aorto-arteritis; pro-thrombotic anatomic abnormalities; and underlying coagulation disorders (1,19). Moreover, the cardiac systolic function is frequently reduced in most patients, which predisposes them to blood stasis, inflammatory changes and hypercoagulability (Virchow's triad). This hypercoagulative state is further promoted by the sustained hypertension, an increased platelet aggregation and the vascular endothelial injury evidenced in these neuroendocrine tumors, all of which prompt the patient to form thrombi in low flow areas. Some authors argue that the most likely underlying etiology is vasospasm, especially in patients with arterial thrombosis without evidence of a cardiac thrombus. Our patient was in a hypercoagulable state as a result of excessive norepinephrine release from the paraganglioma. Additionally, the symptoms of heart failure and peripheral arterial insufficiency contributed to the patient remaining at rest for a long time. Likewise, the anatomic location of the tumor compressing the vena cava and probably to a certain degree the abdominal aorta favored vascular stasis. It is probable that the combination of all these factors: systemic arterial embolization, uncontrolled release of extremely high levels of catecholamines and an induced hypercoagulable state prompted by anatomical compression and other cytokines and humoral mediators, were the ones probably related to a greater deterioration of the arterial thrombosis and the intracardiac thrombi formation in our case.

There is an established association of pheochromocytoma and catecholamine-induced dilated cardiomyopathy, left ventricular hypertrophy, Takotsubo cardiomyopathy (14), and myocarditis (10). Acute refractory cardiogenic shock has also been reported (14). These associations are explained by acute and chronic repeated episodes of catecholamine surges that cause left ventricular hypertrophy, myocardial necrosis, focal myofibrillar degeneration, and subsequent fibrosis; all mediated through direct damage of the myocytes or/and indirect ischemic injury as a consequence of microvascular dysfunction (3). Moreover, intracardiac thrombi has been found in a small fraction of patients with pheochromocytoma (6,8,9,12), and in two reported patients with paraganglioma (17,18). The hypercoagulable state induced by high catecholamine levels predisposes patients to form thrombi in low flow areas with a later risk of embolization to brain, kidneys and distal extremities, as might have happened in our case (6,8,9). Even though the presence of an intracardiac pheochromocytoma has been reported previously (21), it is unlikely in our case since the intracardiac mass resolved following anticoagulation.

Systemic arterial embolization, intracardiac thrombi and peripheral arterial thrombosis have been described in several case reports, some of them requiring the need for emergency treatment due to life-threatening conditions (Table 1). Of the 16 patients reported in the literature with arterial thrombosis (including ours), upper limb arterial thrombosis has been reported in 3 (18.8%) (1,5,6), lower limb arterial thrombosis in 5 (31.3%) (8-10,12), thrombosis of cerebral arteries in 8 (50.0%) (1,5,8,12,13,16–18), carotid arteries in 4 (25.0%) (5,7,11,13), renal arteries in 3 (31.3%) (9,15,18), and coronary (14) and innominate (7) arteries in 1 patient (6.3% each). Furthermore, a left ventricle thrombus was identified in only 7 of 12 patients (58.0%) (6,8,9,12,17,18). There are not differences in gender distribution (8 male and 8 female), and age at presentation varied from 13 to 63 years-old, with a mean of 43.9 years-old. As evidenced, lower limb arterial thrombosis is the second most common site for thrombosis in these tumors. In all cases of arterial thrombosis related to paragangliomas, the clinical presentation was due to multiple cerebral embolisms in association of an intracardiac thrombi (17,18), except for Van and cols. (16), which does not specify the presence or absence of an intracardiac thrombi (Table 2). Our patient is the first case that reports lower limb arterial thrombosis and paraganglioma.

**Table 1:** Previously published cases of arterial thrombotic complications of pheochromocytoma and paraganglioma

Study	Pts, No.	Age, years	Sex	Size, cm	Arterial thrombosis as initial presentation before diagnosis of PPGL	Arterial thrombosis location	Clinical presentation	Intracardiac thrombi	Type of tumor	History of active AP/PG	Germline mutation	Treatment*	Long-term outcome
Current	1	21	M	5	Yes	Bilateral femoral arteries	Bilateral lower limb pain and swelling and hypertensive emergency	Yes	PG	No	No family history mentioned	PTAE, IV heparin, warfarin	CR
Vindenes et al. (1)	1	50	F	7.8	No	Right MCA, right axillary and brachial arteries	Hypertensive emergency and renal failure	No	Left AP	No	NS	PTAE, IV heparin	Died
Kaiser et al. (5)	1	50	F	7.8	No	Right subclavian to radial artery, bilateral carotid arteries, right MCA	Ataxia and hypertensive emergency	No	Left AP	Yes	NS	IV heparin, open thrombectomy	Died
Hou et al. (6)	1	47	F	8.0	Yes	Left axillary artery	Acute onset of numbness of her left arm	Yes	Left AP	No	No	Anticoagulation	CR
Raghavan et al. (7)	1	56	M	10	Yes	Right innominate and left common carotid arteries	Stroke	NS	Malignant Right AP	NS	NS	NS	NS
Heindel et al. (8)	1	49	M	10	Yes	Cerebral and right femoral arteries	Pneumonia	Yes	Left AP	No	NS	IV heparin, aspirin	CR
Zhou et al. (9)	1	43	F	7	Yes	Renal and bilateral femoral arteries	Dyspnea, tachycardia and vomiting	Yes	Right AP	No	NS	LMH, warfarin. PTAE and LAFL in femoral artery	Bilateral high level below the knee amputation. CR after tumor removal
Battimelli et al. (10)	1	63	F	7	Yes	Bilateral tibial arteries	Resting dyspnea, precordial pain and peripheral coldness	No	Left AP	No	NS	PTAE	NS
Ueda et al. (11)	1	44	F	3.5	Yes	Left ICA	Stroke: Gait disturbance and loss of appetite	No	Left AP	No	NS	IV heparin, aspirin	CR
Dagartzikas et al. (12)	1	13	M	8.5	Yes	Right MCA, bilateral lower limb arteries	Stroke: Confusion, anxiety, diaphoresis and moderate distress	Yes	Malignant Left AP	No	NS	Thrombolysis (TPA), LMH, bilateral lower extremity PTAE	Left-side hemiparesis. Referred for chemotherapy
Hill and Schwartzman (13)	1	54	F	NS	Yes	Right MCA, right and left ICA	Stroke: Left-sided hemiparesis and gastrointestinal bleeding	NS	Right AP	No	NS	IV heparin	Died
Cheaito (14)	1	55	M	6.5	Yes	Coronary	Chest pain	No	AP, side NS	No	NS	PTAE and primary stenting	NS
Thewjitcharoen et al. (15)	1	47	F	NS	No	Renal	Right retroperitoneal pain	NS	Right AP	No	NS	None	NS
Van et al. (16)	1	14	M	5.1	Yes	Cerebral artery (NS)	Stroke: Left-side limb weakness and fascial palsy	NS	Retroperitoneal PG	No	No family history mentioned	None	CR
Shafiq et al. (17)	1	47	M	5.3	No	Vertebral basilar and cerebral arteries (embolisms)	Chest pain and palpitations	Yes	Mediastinal PG	No	NS	Aspirin, IV heparin	CR
Buchbinder et al. (18)	1	49	M	NS	Yes	Renal, multiple cerebral arteries (embolisms)	Flu-like symptoms, left upper quadrant abdominal pain and constipation	Yes	Retroperitoneal PG	No	NS	Heparin, warfarin, enoxaparin	CR

F: female; M: male; PPGL: pheochromocytoma and paraganglioma; PG: paraganglioma; AP: adrenal pheochromocytoma; PTAE: percutaneous transluminal angioplasty/embolectomy; LAFL: local intra-arterial fibrinolysis; MCA: medial cerebral artery; ICA: internal carotid artery; TPA: tissue plasminogen activator; LMH: low molecular heparin; IV: intravenous; NS: not specified; CR: complete recovery.

* Treatment besides surgical removal of the PG/AP.

**Table 2:** Summary of clinical features of arterial thrombotic complications of pheochromocytoma and paraganglioma presented between 1981 and 2019

N		16
Age (years), X±SD		43.9 ± 14.3
Gender (M), n (%)		8 (50.0)
Size (cm), X±SD		7.0 ± 1.9
Arterial thrombosis as initial presentation before diagnosis of PPGL, n (%)		12/16 (75)
Thrombosis number sites, Med (min-max)		2 (1-3)
Arterial thrombosis location, n (%)		
	Cerebral	8 (50.0)
	Lower limb	5 (31.3)
	Renal	3 (31.3)
	Carotid	4 (25.0)
	Upper limb	3 (18.8)
	Innominate	1 (6.3)
	Coronary	1 (6.3)
Intracardiac thrombi, n (%)		7/12 (58.0)
Type of tumor, n (%)		
	Adrenal pheochromocytoma	12 (75.0)
	Paraganglioma	4 (25.0)
Location, n (%)		
	Left Adrenal	7 (58.0)
	Right adrenal	4 (33.0)
	Adrenal, NS	1 (6.3)
	Extra-adrenal	4 (25.0)
History of active AP/PG		1 (6.3)
Treatment, n (%)		
	Heparin	10 (62.5)
	PTAE	6 (37.5)
	Warfarin	3 (18.8)
	Aspirin	3 (18.8)
	Thrombolysis	2 (12.5)
	None	2 (12.5)
	Not specified	2 (12.5)
	Open thrombectomy	1 (6.3)
Long-term outcome, n (%)		
	CR	8 (50.0)
	Died	3 (18.8)
	Referred for chemotherapy	1 (6.3)
	NS	4 (25.0)

M: male; NS: not specified; PTAE: percutaneous transluminal angioplasty/embolectomy; CR: complete remission.

Prompt diagnosis of a catecholamine secreting tumor must be made, especially in cases related with hemodynamic instability and acute arterial thrombosis. As demonstrated in our literature review, a known history of active pheochromocytoma was evident only in one patient (5). Moreover, the presence of arterial thrombosis as the initial manifestation prior to the diagnosis of PPGL was present in 12/16 patients (75%) (6–14,16,18). This showed the importance of considering this type of neuroendocrine tumors in the differential diagnosis of arterial thrombosis, especially when associated with the presence of arterial hypertension.

Since at least one-third of all patients with PPGLs have disease-causing germline mutations, screening for hereditary forms of PPGLs is generally recommended, especially when the diagnosis is made at younger age (as in our case) or in the presence of multifocal disease (2). Additionally, germline genetic forms of PPGLs have been found to be extra-adrenal and recurrent (22). There have been reported around 14 susceptibility genes, which include among the most common those related to multiple endocrine neoplasia type 2, von Hippel Lindau disease, neurofibromatosis type I and familial catecholamine-hypersecreting tumors in succinate dehydrogenase (SDH) gene mutation (2). There have not been reported any associations between germline mutations and the occurrence of arterial thrombosis and PPGLs in previous reports, as evidenced in Table 1. We did not find any evidence of positive family history, syndromic or malignant features nor metastatic disease related to hereditary forms of PPGLs in our case. O the other hand, this was a recurrent paraganglioma that was first detected at the age of 14y and recurred at 21y. Due to the clinical presentation with no metastasis, extra-adrenal and elevated normetanephrine levels, genetic testing for *SDHB*, *SDHD*, *VHL*, *SDHC*, *MAX* might be an option (22). Unfortunately, due to the financial costs of genetic testing in our hospital, molecular diagnosis could not be done. Due to the hypercoagulable state induced by pheochromocytomas, treatment options for the thrombotic event include anticoagulation, mechanical thrombectomy, systemic thrombolytic therapy, trans-catheter regional thrombolysis, pulse-spray pharmaco-mechanical thrombolysis and/or angioplasty (9,12,19). Intravenous heparin and low molecular heparin, with or without percutaneous embolectomy, has been the main treatment recommended in most patients with arterial thrombosis in the presence of a PPGL (1,5,11–13). Even though the outcome is generally favorable, a complete remission of the thrombosis is only achieved when the anticoagulation is followed by the surgical removal of the tumor, as concluded in previous reports (6,8,9,11,16–18), confirming the potential role of the pheochromocytoma in the hypercoagulable state and the risk of thrombotic complications. Even with the appropriate treatment, there are cases in which the clinical presentation is very aggressive, with significant cardiovascular instability, which has culminated with the death of the patients (1,5,13), or the presence of sequelae after surgery (9,12). Anticoagulation and thrombectomy were adequately prescribed since their detection in our case, which might have limited the limb ischemia and necrosis in our patient as well as the disappearance of the cardiac thrombus. A complete remission of the thrombosis, without long-term sequelae, was accomplished after the retroperitoneal tumor was successfully resected.

We conclude that PPGLs are rare neuroendocrine tumors that can sometimes be related to uncommon vascular and thrombotic manifestations. Our case illustrates a rare disease that was initially detected by the presence of symptoms of lower limb ischemia and heart failure. Further investigation is needed to evaluate if patients with PPGL need preventive anticoagulation to avoid further devastating thrombotic complications.
